# The Role of Exosome-Derived microRNA on Lung Cancer Metastasis Progression

**DOI:** 10.3390/biom13111574

**Published:** 2023-10-25

**Authors:** Israel Martinez-Espinosa, José Antonio Serrato, Blanca Ortiz-Quintero

**Affiliations:** Department of Molecular Biomedicine and Translational Research, Instituto Nacional de Enfermedades Respiratorias Ismael Cosío Villegas, Mexico City CP 14080, Mexico; imartinezfq@gmail.com (I.M.-E.); serratoiner@gmail.com (J.A.S.)

**Keywords:** exosome, microRNA, lung cancer, metastasis

## Abstract

The high mortality from lung cancer is mainly attributed to the presence of metastases at the time of diagnosis. Despite being the leading cause of lung cancer death, the underlying molecular mechanisms driving metastasis progression are still not fully understood. Recent studies suggest that tumor cell exosomes play a significant role in tumor progression through intercellular communication between tumor cells, the microenvironment, and distant organs. Furthermore, evidence shows that exosomes release biologically active components to distant sites and organs, which direct metastasis by preparing metastatic pre-niche and stimulating tumorigenesis. As a result, identifying the active components of exosome cargo has become a critical area of research in recent years. Among these components are microRNAs, which are associated with tumor progression and metastasis in lung cancer. Although research into exosome-derived microRNA (exosomal miRNAs) is still in its early stages, it holds promise as a potential target for lung cancer therapy. Understanding how exosomal microRNAs promote metastasis will provide evidence for developing new targeted treatments. This review summarizes current research on exosomal miRNAs’ role in metastasis progression mechanisms, focusing on lung cancer.

## 1. Introduction

Lung cancer (LC) is the leading cause of death from malignant neoplasms in both genders worldwide, with the lowest 5-year survival rate and more than 2.1 million new cases estimated in 2020 [1,2,3,4,5]. Despite recent advances in prevention, detection, and treatment, the high mortality rate of lung cancer prevails, primarily attributable to the presence of metastases at the time of diagnosis. More than half of patients are diagnosed when cancer cells have spread to mediastinal and ipsilateral lymph nodes, brain, bones, and adrenal glands. Moreover, lung cancer patients with metastasis to distant parts of the body have significantly lower survival rates than those with regional or localized cancer. In non-small cell lung cancer (NSCLC), patients with distant metastasis have an 8% 5-year survival rate. In contrast, patients with metastasis in nearby lymph nodes and localized cancer in the lung have a 5-year survival rate of 37% and 63%, respectively [6]. Therefore, understanding the mechanisms of metastasis progression is crucial for improving patients’ survival rates and quality of life.

Recently, exosomes derived from tumor cells have been associated with tumor progression through intercellular communication mechanisms [7,8,9,10]. Furthermore, the evidence suggests that tumor exosomes direct metastasis to distant organs by preparing a metastatic pre-niche, recognizing resident cells [11], and releasing biologically active components that favor the tumorigenic process [12,13,14]. Exosomes are small extracellular vesicles measuring 30–150 nm in diameter released by cells into the extracellular space, including body fluids and circulation. These vesicles contain biologically active molecules such as proteins, nucleic acids, amino acids, lipids, and metabolites. When they reach a target receptor cell, they release these molecules, which have a biological effect on the recipient cell [15,16]. Exosomes constitute an essential mechanism of intercellular communication in homeostasis and diseases, including cancer. The cargo components of the exosomes are selectively incorporated into the vesicle and vary depending on the cell origin and its physiological state [15,16]. Therefore, identifying the active components of exosome cargo has become a critical area of research in recent years. Among the components transported by tumor exosomes are the microRNAs (miRNAs), which are implicated in mechanisms of tumorigenic progression [9,14,17,18,19] and as biomarkers in liquid biopsies in cancer. miRNAs are non-coding small RNA molecules of ~22 long nucleotides that regulate gene expression post-transcriptionally and are involved in essential cellular processes such as development, proliferation, differentiation, and apoptosis [20,21,22,23,24]. MiRNAs regulate gene expression by targeting the 3′ untranslated region (UTR) of the mRNA of the target gene, thereby regulating protein levels [25,26,27]. It was soon discovered that miRNAs are sorted into exosomes, released into the extracellular space, and transferred from the donor cell to recipient cells, where they alter the expression of target genes [28]. This finding expanded the researchers’ perspective of miRNA’s range of action not only as intracellular regulators but also as molecules that could reprogram the behavior of target cells.

In lung cancer, exosome-derived miRNAs (exosomal miRNAs) have gained visibility as potential diagnostic and prognostic biomarkers in liquid biopsies. At the same time, exosomes are being studied as vehicles for delivering miRNA anticancer drugs to the lungs [29,30,31].

Recent research has focused on the role of exosomal microRNA in promoting lung cancer metastasis. Evidence indicates that exosomal miRNAs regulate metastasis-related mechanisms, such as the epithelial–mesenchymal transition (EMT), angiogenesis, migration, and immune response evasion [32,33]. Moreover, recent evidence suggests the participation of exosomal miRNAs in promoting distant metastasis in lung cancer, such as bone metastasis. Understanding how exosomal miRNAs promote cancer spread will be crucial in developing targeted therapies that can significantly improve survival rates and enhance the quality of life of lung cancer patients. This review summarizes current research on exosomal miRNAs’ role in metastasis progression mechanisms, focusing on lung cancer. It also provides an overview of exosome biogenesis and miRNA sorting into exosomes as cargo while emphasizing the identity of the exosomal miRNAs implicated in metastasis mechanisms, the donor cell, recipient cells, and molecular targets.

## 2. Exosome Biogenesis and miRNA Sorting

### 2.1. Biogenesis of Exosomes

**Figure 1:**
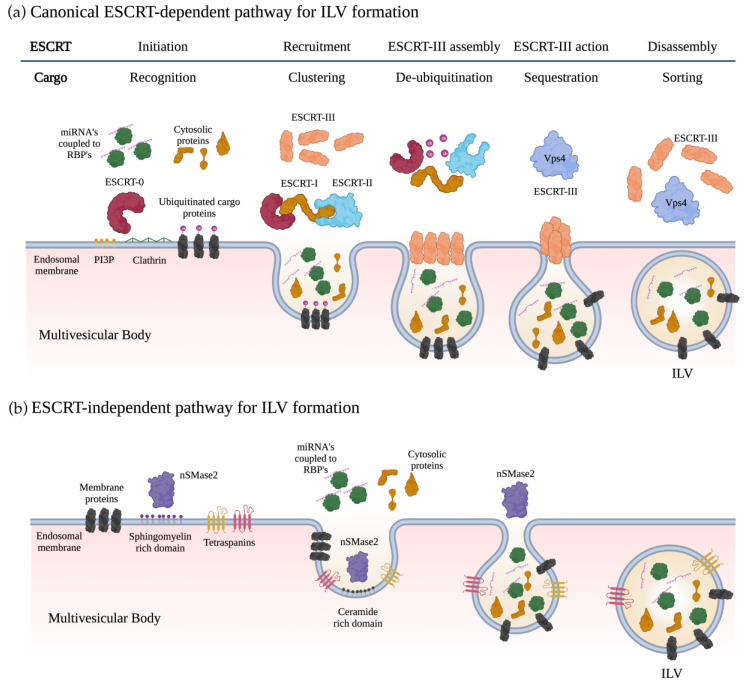
Exosome biogenesis. (**a**) The ESCRT-dependent pathway begins by binding the ESCRT-0 complex to the endosomal membrane. This is achieved by recognizing Phosphatidylinositol 3P (PI3P) domains and ubiquitinated proteins coupled to clathrin on the surface of the endosome. Once anchored, ESCRT-0 recruits the ESCRT-I complex, which then recruits the ESCRT-II complex. The coordinated action of ESCRT-I and II induces the formation of a pouch by invaginating the endosomal membrane. This pouch sorts and packages ubiquitinated proteins, cytosolic proteins, and miRNA-RBP complexes inside. ESCRT-I then recruits the different components of the ESCRT-III complex over the neck of the pouch. The ESCRT-III complex folds over the neck of the nascent ILVs and releases it from the endosomal membrane. Finally, the ESCRT-III complex components are disassembled by the Vps4 enzyme and its accessory protein, LIP5. (**b**) In the ESCRT-independent pathway, sphingomyelin hydrolysis occurs in the endosomal membrane through the nSMase2. This results in the formation of lipid raft microdomains, which prompt the endosomal membrane to fold inward, forming ILVs. During this process, cytosolic proteins, membrane proteins, and miRNA-RBP complexes are enclosed within the ILV. These ILVs, abundant in ceramide, are formed naturally and do not require ESCRT machinery. ILV, intraluminal vesicles; RBP, RNA binding protein; Vps4, vacuolar protein sorting 4 homolog A; LIP5, Lipase 5; nSMase2, neutral enzyme sphingomyelinase 2. Created with BioRender.com, “https://www.biorender.com/ (accessed on 4 September 2023)”.

Exosome biogenesis begins with the cytoplasmic membrane inwardly budding to create early endosomes. During this process, these endosomes incorporate membranal proteins, lipids, and soluble proteins associated with the extracellular milieu into cells. In addition, the trans-Golgi network (TGN) and endoplasmic reticulum (ER) can contribute to endocytic cargo and luminal constituents in the early endosomes [16]. The early endosomes mature into late endosomes. Within the late endosomes, intraluminal vesicles (ILVs) are created by the invagination of the endosomal membrane, where cytosolic proteins, nucleic acids, and lipids are sorted. Late endosomes containing multiple ILVs are named multivesicular bodies (MVBs) [34]. The MVB can either fuse with lysosomes and autophagosomes for degradation or fuse with the plasma membrane to release ILVs as exosomes [35,36]. ILV formation requires the endosome membrane to become highly enriched for tetraspanins such as CD9 and CD63 [37]. It also requires the recruitment of the endosomal sorting complexes required for transport (ESCRT) to the site of ILV formation. ESCRT is a multiprotein complex that enables vesicle budding and cargo sorting into MVBs. ESCRT consists of four different protein complexes: ESCRT-0, -I, -II, -III, and the associated AAA ATPase Vps4 complex [30]. A subunit of ESCRT-0, the hepatocyte growth factor-regulated tyrosine kinase substrate (HRS), recognizes and sorts ubiquitinated cargoes to phosphatidylinositol 3-phosphate (PI3P)-enriched endosomal compartments [38]. PI3P is a phospholipid in early and late endosomes that regulate cell signaling and membrane trafficking [39]. Subsequently, HRS binds to the ubiquitin E2 variant (UEV) domain of the TSG101 protein from the ESCRT-I complex, linking both complexes. The sequential binding of ESCRT-I and ESCRT-II complexes promotes the accumulation of other lipids (e.g., ceramide and sphingosine) around clusters of ubiquitinated proteins, forming a membrane pouch. Then, the charged multivesicular body protein subunit (CHMP6) of ESCRT-III binds to the ESCRT-II complex and recruits CHMP4 (Snf7), which polymerizes, forming a coiled neck around the membrane pouch [40]. After adding CHMP3, the bud is cleaved to form ILVs, followed by the dissociation of the ESCRT machinery and recycling through ATP catalysis by Vps4 [15,41,42] (Figure 1a).

An ESCRT-independent lipid-mediated pathway occurs in various biological processes, including RNA viral transfer and the invasive process of cancer cells [43,44]. In this mechanism, complex lipids, such as ceramides, membrane sphingolipids, and sphingomyelinases, can self-associate to form raft-like structures, which induce curvature in the membrane, leading to inward budding for ILV formation [45] (Figure 1b). It has been shown that the nSMase2 enzyme is critical for this mechanism, as well as Rab31 GTPase and tetraspanin CD63 [46].

### 2.2. Loading of miRNAs into Exosomes

Accumulative evidence indicates that miRNAs are selectively packaged into EVs, actively released, and delivered functionally into recipient cells. However, the mechanisms by which miRNAs are loaded into exosomes are still under investigation. Studies suggest that sorting occurs via various processes, possibly dependent on the lineage and physiological state of donor cells and the EV origin.

Most studies focus on the mechanisms of sorting miRNA into EVs since current methods are ineffective at isolating pure subpopulations. Several potential sorting mechanisms have been proposed [47,48]: (a) the neural sphingomyelinase 2 (nSMase2)-mediated mechanism; (b) the sumoylated heterogeneous nuclear ribonucleoproteins (hnRNPs)-mediated mechanism; (c) other RNA-binding proteins-mediated miRNA-sorting mechanism; and (d) the Argonaute 2 (AGO2)-mediated mechanism. These mechanisms are based on the following research works: Kosaka et al. [49] found that inhibiting the activity of nSMase2 with the inhibitor GW4869 and small interfering RNA led to a decrease in the secretion of miRNAs. Conversely, overexpressing nSMase2 increased extracellular miRNAs. In a later report [50], the researchers revealed that the overexpression of nSMase2 in murine breast cancer cells led to an increase in the number of exosomes and exosomal miRNAs miR-16 and miR-210, while inhibiting nSMase2 had the opposite effect. Villarroya-Beltri et al. [51] found that sumoylated hnRNPA2B1 (an RNA-binding protein; RBP) recognizes the GGAG motif in the 3´ portion of certain miRNA sequences and controls their loading into the exosomes in human T-cells. Similarly, Santangelo et al. [52] reported that the RBP called synaptotagmin-binding cytoplasmic RNA-interacting protein (SYNCRIP or hnRNPQ) recognizes miRNAs containing the motif GGCU via the Syncrip’s RNA-recognition motif (RRM) domains and selectively sorts them into the exosomes of hepatocytes. Further, Hobor et al. [53] discovered that SYNCRIP/hnRNPQ has an additional sequence-specific RNA-binding domain called NURR in its amino-terminal domain that mediates the specific recognition of short sequences in miRNA targets. The NURR domain is also connected to Syncrip’s RRM domains via a non-canonical structural element, resulting in high-affinity miRNA binding. Syncrip’s selection of target miRNAs requires recognition of the miRNA sequence by the NURR domain and binding of the RRM domains 5’ to this sequence. This unique structural arrangement enables Syncrip to select miRNAs with different seed sequences. Several other RBPs, including Y-box binding protein 1 (YBX-1) [54,55], MEX3C [56], major vault protein (MVP) [57], and La protein [58], have been associated with miRNA-sorting mechanisms. McKenzie et al. [59] found that increased KRAS activity caused by mutation inhibits the localization of AGO2 to MVEs and decreases AGO2 secretion in exosomes. This event regulates the levels of three miRNAs (let-7a, miR-100, and miR-320a) present in exosomes from colon cancer cells, suggesting that AGO2 is involved in a loading mechanism.

### 2.3. Exosome Incorporation and Cargo Processing in the Recipient Cells

The exact mechanism of how exosomes interact with the plasma membrane of recipient cells and uptake and transfer their contents is not yet fully understood. Moreover, studies on the subject focus on EVs instead of the exosome subtype. Research indicates that EVs contact the recipient cell via different receptors at the plasma membrane. This interaction led to intracellular signaling events, which later directed the process of exosome internalization into the cell. Several surface molecules have been associated with the first interaction of exosome-recipient cells, and their diversity is likely dependent on the type of EVs and recipient cells. Molecules such as tetraspanins, sulfate proteoglycans, lectins, integrin receptors, and tetherin have a role in docking EVs to recipient cells [51,60,61,62]. In cancer, it was revealed that EVs can express specific receptors that selectively target cell types, locally and at a distance, allowing them to exhibit organotropism. For instance, evidence in vivo showed that tumor-derived EVs expressing unique integrins showed organotropism to the lung (α6β4 and α6β1) and the liver (αvβ5) [11]. Another example is the amyloid precursor protein (APP) on exosomes derived from neuroblastoma cells specifically targeting neurons. However, data also indicate non-selective binding mechanisms, such as interactions via phosphatidylserine (PS) on EV membranes [63,64]. Once EVs are attached to the recipient cell, they can be taken up through various endocytosis pathways, such as clathrin-mediated or clathrin-independent endocytosis, micropinocytosis, phagocytosis, or endocytosis via caveolae and lipid rafts [61,65,66]. After the recipient cells take up EVs, they follow the endocytic pathway and reach the multivesicular endosomes (MVEs), which may be directed to the lysosome. If EVs are not digested, they release their contents into the cytoplasm of the recipient cell [67]. Currently, there is limited knowledge regarding the intracellular processing of EVs and how the recipient cell interprets delivered instructions.

## 3. General Mechanisms of Lung Cancer Metastasis

Metastasis is a process that involves the dissemination of cancer cells from the primary tumor site to other parts of the body. For cancer cells to spread to other parts of the body, they need to break away from the primary tumor, travel through the bloodstream or lymphatic system, withstand migration through circulation, adapt to a new environment in the secondary site and colonize it, and evade the immune response system [68]. Metastasis occurs in more than half of lung cancer patients. Lung cancer is diagnosed when cancer cells have already spread to the mediastinal and ipsilateral lymph nodes, brain, bones, liver, and adrenal glands [69,70]. Among those body sites, distant extrathoracic metastasis is the primary cause of lung cancer-related deaths, with the bone, brain, and liver being the most common sites for metastases in all histological subtypes of non-small cell lung cancer (NSCLC) [71,72]. Meanwhile, small cell lung cancer (SCLC) typically metastasizes to the liver and bones [73].

**Figure 2:**
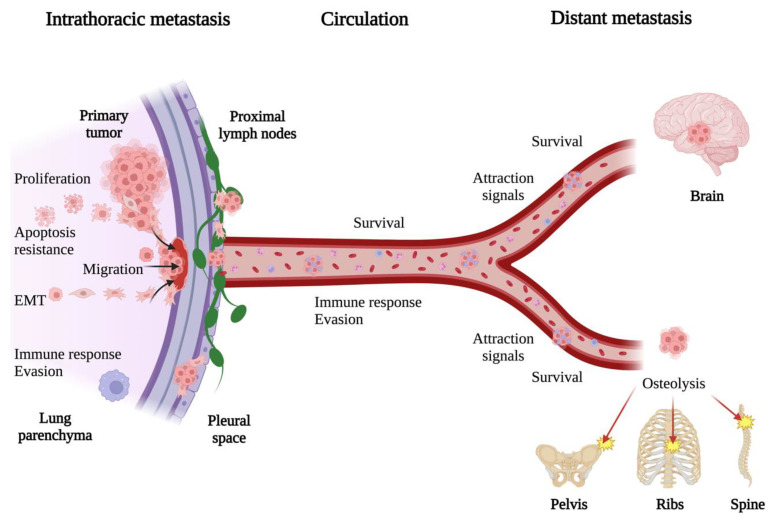
General mechanisms of lung cancer metastasis. The process of lung cancer metastasis includes transforming tumor cells through EMT, their detachment from the ECM, migration to nearby tissue, and spreading throughout the body through blood or lymphatic vessels as CTCs. Lung cancer CTCs can migrate as single cells or clusters and frequently travel surrounded by platelets and neutrophils to evade the host immune response. When CTCs reach a suitable microenvironment, they attach and form a new metastatic focus in different body parts, such as the pleural membrane, lymph nodes, bones, and the brain. CTCs, circulating tumor cells; ECM, extracellular matrix; EMT, epithelial-to-mesenchymal transition. Created with BioRender.com, “https://www.biorender.com/ (accessed on 4 September 2023)”.

Regardless of their origin and organ spreading, the steps in cancer metastasis are similar in different cancer types. The steps of the metastatic process include (a) the detachment of cancer cells from the extracellular matrix (ECM), (b) invasion and migration into the nearby stroma, (c) entry into circulation through blood or lymphatic vessels, (d) the survival migration of cancer cells through circulation, and (e) the attachment to and invasion of a new body site, forming a new metastatic lesion (Figure 2). Several critical mechanisms are essential for this sequence of events to occur. Angiogenesis is necessary to maintain cancer cell growth in both primary and metastatic tumor sites, while the disruption of the extracellular matrix by several proteolytic enzymes permits cancer cells to migrate through it. In addition, cancer cells undergo epithelial-to-mesenchymal transition (EMT) to detach from the ECM and migrate. Cells undergo EMT; lose desmosomes, adherence junctions, and apical-basal polarity; reshape the cytoskeleton; reduce the expression of E-cadherin; secrete increased enzymes that degrade ECM; and increase the expression of vimentin and N-cadherin, acquiring a motile and invasive phenotype [74]. Once the cancer cells reach and travel into circulation, they are named circulating tumor cells (CTCs). CTCs must withstand intense mechanical flow, immunosurveillance, and oxidative stress during migration through circulation. CTSs often associate with platelets to evade immunosurveillance [75] and undergo reversible metabolic changes to increase their resistance to oxidative stress [76]. CTCs can migrate as single cells or clusters; however, the latter is more likely to survive and form metastases [77,78]. In lung cancer, SCLC seems to migrate as single cells and small clusters of cells in the blood and lymphatic vessels, while NSCLC adenocarcinomas seem to migrate as both small-cell clusters and large migrating complexes of cells [69]. After surviving migration in circulation, cancer cells must be able to adhere to the metastatic focus and adapt to the new microenvironment to colonize it. Research suggests that the colonization of secondary distant sites by CTCs is not a random or passive process. Rather, it depends on certain conditions in the target organs’ microenvironment, in addition to the ability of cancer cells to reach, survive in, and proliferate efficiently in distant organs [79]. Furthermore, how cancer cells spread and grow in distant sites involves communication between resident stromal, immune, and cancer cells. This intercellular communication happens through direct physical contact between cells or soluble factors secreted locally or transported through small extracellular vesicles in the body, such as exosomes [32]. Furthermore, evidence shows that exosomes may direct distant metastasis by preparing metastatic pre-niche and stimulating tumorigenesis [32,80,81].

In the following sections, we will describe how exosomal miRNAs regulate mechanisms related to the progression of lung cancer metastasis, such as EMT and angiogenesis. We will also explore the miRNAs linked to the distant metastasis of organs primarily impacted by the disease, such as the brain and bones.

## 4. Exosomal miRNAs in Lung Cancer EMT

EMT is a natural process during embryogenesis, tumor progression, and metastasis. EMT is a reversible process that allows immotile and tightly bound epithelial cells to differentiate into mesenchymal cells, which possess plasticity and motility. During cancer, EMT can aid in entering cells into the bloodstream. However, once the cells leave the bloodstream and enter a new organ, reversing this state (mesenchymal-to-epithelial transition or MET) enables them to establish a foothold and grow. There are various pathways involved in EMT, including TGF-β, Wnt, Notch, NF-κB, Hedgehog, FGFR, and STAT3, which are regulated by genetic, epigenetic, and microenvironmental factors. One of the epigenetic factors is miRNAs, which have been shown in multiple studies to impact metastasis in lung cancer by either promoting or inhibiting EMT [82,83,84,85,86]. Furthermore, several tumor-derived exosomal miRNAs have been shown to affect lung cancer metastasis by promoting EMT. For example, Yu et al. [87] demonstrated that miR-31-5p-enriched exosomes from A549 and H1299 cells cultured under hypoxic conditions enhanced the migration and invasion of the same cells under normoxic conditions. Exosomal miR-31-5p directly targets the special AT-rich sequence-binding protein 2 (SATB2), which is known to reverse the epithelial–mesenchymal transition. This significantly increases MEK/ERK signaling activation and ultimately contributes to tumor progression in both in vitro and murine xenograft models.

In another study, Wang et al. [88] found that stem-like cells that grow spherical, derived from A549 cells, release exosomes that promote the migration and invasion of A549 and NCI-H1703 lung cancer cells. This is accompanied by the increased expression of N-cadherin, vimentin, MMP-9, and MMP-1, while E-cadherin is downregulated. The dual-luciferase reporter assay confirmed that miR-210-3p targets fibroblast growth factor receptor-like 1 (FGFRL1). When FGFRL1 was silenced in lung cancer cells, their ability to migrate and invade increased, as well as the expression levels of EMT markers N-cadherin and vimentin. Conversely, overexpressing FGFRL1 inhibited these traits. These findings indicate that exosomal miR-210-3p could promote the migration and invasion of lung cancer cells by regulating EMT via FGFRL1 targeting.

Similarly, Hisakane et al. [89] reported an EMT promoter role of exosomal miR-210-3p. In this study, lung cancer cells resistant to Osimertinib HCC827-OR could transfer miR-210-3p in exosomes, causing EMT changes and resistance in parental cells HCC827. Although the transfer of exosomes was not confirmed in this study, there was an increased expression of miR-210-3p in the exosome-treated HCC827 cells. The transfection of miR-210-3p mimics in HCC827 cells led to EMT changes, such as lower levels of vimentin and E-cadherin and increased resistance to Osimertinib, supporting the role of miR-210-3p in EMT regulation.

On his part, He et al. [90] found that exosomes derived from highly metastatic NSCLC cells (SPC-A-1BM) increased the EMT, proliferation, and migration of parental SPC-A-1. They discovered that miR-499a-5p was upregulated in exosomes from SPC-A-1BM and that knocking down miR-499a-5p in SPC-A-1BM cells prevented the effects of exosomes on metastatic-related abilities. Transfection of A549 and SPC-A-1BM cells with miR-499a-5p mimics showed increased EMT, proliferation, and migration of transfected cells via the mTOR pathway with the phosphorylation of S6K1 and BP1 proteins. In addition, when A549 cells were injected into BALB/c nude mice, those receiving miR-499a-5p mimic injections under the skin near tumors developed larger tumor nodules than those receiving the negative control treatment, which suggests an association with tumor progression.

On the other hand, the miR-200 family of endogenous miRNAs, comprising miR-200a/b/c, miR-141, and miR-429, plays a vital role in controlling EMT by acting as an inhibitor. The endogenous miR-200 blocks the production of transcription factors ZEB-1 and ZEB-2, which directly affects the expression of E-cadherin, thereby preserving the epithelial phenotype of epithelial cells [91,92,93,94]. Accordingly, Liu et al. [95] found that cancer-associated fibroblasts (CAFs) transfected with miR-200 mimics secrete exosomes that inhibit the migration, invasion, and EMT of A549 and NCI-H460 lung cancer cells. The effect of miR-200-enriched exosomes was through the inhibition of zinc finger E-box binding homeobox 1 (ZEB1). Exosomes secreted by tumor tissue CAFs had lower miR-200 levels than those secreted by normal fibroblasts, potentially explaining the positive role of CAFs in tumor progression in the tumor microenvironment.

Liu et al. [96] also reported that exosomal miRNAs inhibit EMT. In this study, exosomes from A549 cells induced migration, invasion, and EMT markers in BEAS-2B epithelial lung normal cells. However, they observed that let-7c-5p and miR-181b-5p were decreased in A549 cell-derived exosomes. When exosomes from A549 cells transfected with mimics let-7c-5p and miR-181b-5p were used, there was a decrease in migration and invasion properties on BEAS-2B cells, although evidence of exosome uptake was not provided. Bioinformatic analysis suggested that let-7c-5p and miR-181b-5p may inhibit the EMT process by affecting the mitogen-activated protein kinase (MAPK) signaling pathway, but this was not experimentally tested.

Table 1 summarizes the exosomal miRNAs associated with EMT in lung cancer. The revised literature showed that exosomal miRNAs can function as either promoters or inhibitors of EMT, consistent with information on endogenous miRNAs. The research also revealed no overlap in the molecular targets and pathways of the exosomal miRNAs examined in these studies. This may be due to the limited number of current studies on the topic.

## 5. Exosomal miRNAs in Lung Cancer Angiogenesis

As the primary tumor expands, the tumor microenvironment (TME) becomes more heterogeneous regarding cell types, structure, and metabolic processes [97,98,99]. The growing tumor requires more oxygen and nutrients to keep growing. When it grows beyond a certain size, the blood vessels around it can no longer provide enough oxygen, resulting in hypoxia in various tumor zones [100,101]. This condition promotes angiogenesis to form new blood vessels from existing ones by activating different signaling pathways. Tumor angiogenesis initiates when pro-angiogenic factors, such as the vascular endothelial growth factor (VEGF), the fibroblast growth factor (FGF), and platelet-derived growth factors (PDGFs), stimulate endothelial cells that are typically dormant during homeostasis [102]. Although homeostatic angiogenesis is a well-coordinated process, the continuous overproduction of angiogenic factors during tumor-induced angiogenesis results in structurally abnormal tumor vessels. Tumor vessels are tortuous and dilated, which causes an increase in vascular permeability and high interstitial pressure. This can lead to reduced blood perfusion and increased hypoxic conditions within the tumor microenvironment, resulting in hypoxia, glucose starvation, and immune cell infiltration, which may promote metastasis [103,104].

Among the molecular factors regulating angiogenesis in tumors, studies have shown that specific miRNAs in tumor-derived exosomes may promote lung cancer metastasis by inducing angiogenesis. A study conducted by Fan et al. [105] found that exosomes from A549 and H460 lung cancer cells triggered the CAF phenotype of the NIH/3T3 fibroblast and the secretion of pro-angiogenic factors VEGF-A, FGF2, and MMP9. Moreover, the culture supernatant from exosome-treated NIH/3T3 cells increases the in vitro proliferation, migration, and tube formation of mouse MS-1 endothelial cells. In the in vivo model, exosome-treated NIH/3T3 cells increased microvessel density (MVD) in mice. Lung cancer cell-derived exosomes have high levels of miR-210, and further use of exosomes from cells transfected with miR-210 mimic or anti-miR-210 resulted in the observed and opposite effect on NIH/3T3 cells, supporting its role in angiogenesis. Exosomes overexpressing miR-210 induced the phosphorylation of STAT3 and JAK2 and reduced levels of Ten-eleven translocation 2 (TET2) in NIH/3T3, identified as the target of miR-210. TET2 is a methylcytosine dioxygenase that modulates the hematopoietic stem cell expansion and function by controlling DNA methylation, and Tet2-deficient mice show an increased tumor vasculature model of lung cancer [106,107]. However, the role of a potential regulatory axis, exosomal miR-210-TET2-angiogenesis, should be further studied.

Mao et al. [108] discovered that SCLC patients had higher levels of miR-141 in their plasma and serum exosomes. This correlated with larger tumor size, distant metastasis, and TMN stage, indicating a potential association with cancer progression and metastasis. In vitro experiments showed that exosomes from miR-141-mimic-transfected SCLC cells enhanced human umbilical vein endothelial cell (HUVEC) proliferation, migration, and tube formation. They also enhanced the number of microvessels sprouting from mouse aortic rings in vitro. MiR-141 exosomes injected with Matrigel into BALB/c nude mice subcutaneously increased blood vessels and CD31 expression in the Matrigel plugs, indicating their ability to promote neovascularization. miR-141 targets the Krueppel-like factor 12 (KLF12), and knocking down KLF12 led to increased HUVEC proliferation tube formation and microvessel sprouting from aortic rings. This suggests that miR-141 promotes angiogenesis by targeting KLF12 on HUVEC. Krueppel-like factors are transcription factors that play a significant role in various cellular processes, including proliferation, apoptosis, and migration [109]. In endometrial and breast cancer, the overexpression of KLF12 has been linked to tumor growth and proliferation [110,111]. However, its role in lung cancer is still being investigated.

Similarly, Wang et al. [112] found that tumor-derived exosomal miRNA-141 promotes angiogenesis and lung cancer progression in NSCLC, however, by targeting the growth arrest-specific homeobox gene (GAX). In this study, miRNA-141 mimics were transfected in A549 lung adenocarcinoma cells, and their exosomes enhanced the migration and invasion of non-transfected A549, as well as the proliferation and the formation of tubes of HUVEC. Through the luciferase assay, the authors showed that miR-141 targets GAX in HUVEC, and the exosomes obtained from miR-141 transfected cells resulted in reduced levels of GAX in HUVEC. It is known that the GAX gene is a negative transcriptional regulator of endothelial cells that is downregulated by angiogenic factors [113]. Still, the regulatory role of exosomal miR-141 on GAX expression in lung cancer should be further explored.

In another study, Chang et al. [114] discovered increased levels of miR-197-3p in tumor tissue and serum from lung adenocarcinoma patients with metastasis, which was also linked to a poorer prognosis. In vitro experiments showed that miR-197-3p-transfected A549 and H1299 cells stimulated the proliferation, migration, and tube formation of HUVEC, while transfection with miR-197-3p inhibitors had the opposite effects. They identified the tissue inhibitors of metalloproteinase 2 (TIMP2) and 3 (TIMP3) as target genes of miR-197-3p in HUVECs. Further, in vivo experiments supported that exosomes overexpressing miR-197-3p promoted the proliferation, migration, and tube formation of HUVEC cells and lung metastasis via TIMP2/3-mediated angiogenesis.

According to Ma et al. [115], higher levels of exosomal miR-3157-3p were found in the plasma of NSCLC patients compared to healthy controls. They found that exosomes from miR-3157-3p-transfected A549 and H1299 NSCLC cells induced the proliferation, migration, and vascular permeability of HUVEC cells by downregulating TIMP2 and Kruppel-like transcription factor 2 (KLF2). Mechanistically, the downregulation of TIMP2/KLF2 increased the expression of VEFG, MMP2, and MMP9 and decreased the expression of ZO-1, occluding, and claudin-5, promoting angiogenesis and increased vascular permeability. In a xenograft murine model, they observed that treatment with miR-3157-3p-exosomes resulted in higher microvessel density and larger tumor size, increased expression of VEFG and MMP2, and decreased expression of TIMP2 and KLF2 compared to the control treatment. Finally, they observed that injection in the tail vein of exosomes derived from transfected A549 cells increased the number of lung metastatic nodules in comparison with control exosomes.

Shao et al. [116] proposed that exosomal miR-494-3p regulates angiogenesis through the c-Jun and the phosphatase and tensin homolog (PTEN) pathways. Their experiment used a specific Jun N-terminal kinase (JNK) inhibitor SP600125 or CRISPR/Cas9 to delete c-Jun. They found that exosomes from SP600125-treated A549 cancer cells or c-Jun-KO-A549 cells showed decreased miR-494-3p levels while inhibiting tube formation, increased PTEN levels, and decreased Akt phosphorylation in HUVEC cells. In vivo, matrigel plugs assays showed that exosomes from c-Jun-KO cells suppressed stimulated vascularization. The transfection of HUVEC cells with miR-494-3p agomirs promoted tube formation and downregulated PTEN levels, suggesting a role of this miRNA in the observed effects. Although PTEN has binding sequences for miR-494-3p, the study did not directly demonstrate the targeting of PTEN by miR-494-3p.

In accordance, Kim et al. [117] also proposed that exosomes originating from A549 cells can enhance tumor angiogenesis and metastasis. Their research showed that miR-610-5p-enriched exosomes derived from A549 cells increased tube length and migration in HUVEC cells while reducing levels of RCAN1.4, which is a direct target of miR-610-5p. The study found that when miR-610-5p was overexpressed in NSCLC cells, it increased the metastatic potential of NSCLC cells injected into mice’s lateral tail veins. On the other hand, the suppression of RCAN1.4 enhanced the characteristics of these cells in vitro. These results suggested that miR-619-5p-3p plays a crucial role by inhibiting the tumor suppressor RCAN1.4, affecting the proliferation and metastasis of NSCLC. In addition, this study found increased expression of miR-619-5p, decreased expression of RCAN1.4, and increased expression of CD31 in the tumor tissue of NSCLC patients, suggesting their role in tumor promotion and angiogenesis.

The reviewed literature showed that six different exosomal miRNAs can potentially promote angiogenesis. However, only miR-210 and miR-141 were shown to promote microvessel density in vivo. Additionally, only miR-197-3p was observed to affect tumor growth in vivo through its impact on angiogenesis. Mechanistically, two miRNAs, miR-197-3p and miR-3157-3p, target TIMP2, but the exact pathways involved are still unknown. TIMP2 is known to inhibit protease activity in tissues and suppress the proliferation of quiescent tissues in response to angiogenic factors. There were no further coincidences. Table 2 summarizes the exosomal miRNAs associated with angiogenesis in lung cancer.

## 6. Exosomal miRNAs in Other Mechanisms Affecting Lung Cancer Metastasis

Before spreading to surrounding tissues and separating from the original site to form metastases, tumor cells undergo various processes that support the progression of the disease. These mechanisms include increased proliferation, migration, resistance to programmed cell death, and avoidance of the local immune system. When circulating, tumor cells must avoid the body’s immune response and successfully reach the secondary tumor site. After reaching the new tumor site, they must adjust to the microenvironment by activating survival signals, promoting cell growth, developing new blood vessels, and avoiding the local immune response. Thus, these mechanisms that facilitate cancer development also impact metastasis progression. Tumor exosomal miRNAs also influence lung cancer metastasis by negatively or positively regulating these mechanisms.

### 6.1. Regulation of Immune Response

To fight tumors, the immune system must activate T lymphocytes and antigen-presenting cells (APCs) such as macrophages and dendritic cells (DCs) to target tumor antigens. The APCs present tumor antigens through the MHCI or II-peptide complex, which activates CD8+ cytotoxic and CD4+ helper T-cells. These T-cells must move into stromal tissue to reach the tumor. Once they reach the tumor, the CD4+ T-cells release cytokines like IFNγ and TNF to enhance the cytotoxic CD8+ T-cell-mediated response, strengthening the antitumor immune response [69,118]. The concomitant infiltration of CD4+ and CD8+ T-cells has been shown to indicate favorable prognoses in NSCLC patients [119]. In addition to T-cells, natural killer cells (NK cells) and macrophages infiltrate the tumor stroma. IFN-γ induces the M1 phenotype of the tumor-associated macrophages (TAMs), contributing to the antitumor response. In general, lung cancer cells evade the immune response by blocking crucial steps in generating a cytotoxic T-cell response. For example, NSCLC tumor cells secrete IL-10, TGFβ, and chemokine CCL20, which promote the proliferation, maturation, and recruitment of regulatory T-cells (Tregs) that suppress CD8+ T-cell-mediated cytotoxic killing [120,121]. Tumor cells secrete TGF-β and prostaglandin E2 (PGE2) to induce DCs to differentiate into regulatory DCs, which inhibit the immune response by secreting cytokines such as IL-10 [122]. Other known mechanisms involve the induction of the M2 phenotype of TAMs, which promote tumor growth by enhancing proliferation and suppressing antitumor immunity in NSCLC [123]. Exosomal miRNAs are among the diverse intercellular communication mechanisms between immune and tumor cells that determine the balance of antitumor and protumor immune responses. Although evidence of lung cancer is still scarce, lung tumor cells secrete exosomal miRNAs that impact immune response components to favor tumor progression.

For example, Li et al. [124] showed that hypoxic stress in NSCLC tumor cells suppresses tumor-secreted exosomal miR-101, which stimulates IL1A and IL6 expression in macrophages via targeting cyclin-dependent protein kinase 8 (CDK8) and SUB1 regulator of transcription (SUB1). The overexpression of miRNA-101 or enriched exosome uptake by macrophages suppresses IL1A and IL6 expression by targeting CDK8, whereas injecting miR-101 into xenografted tumors reduced growth and macrophage tumor infiltration in vivo. These findings suggest that tumor exosomal miR-101 negatively regulates the activation of macrophages in the tumor microenvironment, promoting tumor progression, whereas tumor hypoxia favors inflammation.

According to a study by Donzelli et al. [125], miR-574-5p in small extracellular vesicles (sEVs) negatively regulates the biosynthesis of the pro-inflammatory lipid mediator prostaglandin E2 (PGE2). The study discovered a feedback mechanism in which endogenous miR-574-5p in A549 and 2106T cells promotes the biosynthesis of PGE2, which in turn induces the sorting of miR-574-5p in sEVs through EP1/3 receptors. The sEV-derived miR-574-5p then reduces the levels of pro-inflammatory PGE2 in A549 cells but not in 2106T cells. This regulation relies on the activation of TLR7/8 in A549 (lung adenocarcinoma) cells but not in 2106T (squamous cell carcinoma) cells.

Ma et al. [126] found that miR-181b was upregulated in exosomes from NSCLC cells and serum from NSCLC patients. Tumor exosomal miR-181b was transferred into macrophages, enhancing macrophage M2 polarization and diminishing inflammation. Consequently, the conditioned medium from macrophages treated with exosomes-derived NSCLC cells promotes NSCLC cell proliferation, migration, and invasion. This regulation depends on the activation of JAK2 and STAT3.

On the contrary, Liu et al. [127] reported that exosomal miR-770, derived from NSCLC tumor cells, inhibits the polarization of M2 macrophages, consequently favoring inflammation. This regulation depends on the downregulation of MAP3K1. The authors found that the exosomal miR-770 significantly curbs the growth of NSCLC tumors in vivo by obstructing M2 macrophage polarization. Accordingly, miR-770 was downregulated in NSCLC tissue compared to adjacent normal tissue. The findings suggest that exosomal miR-770 plays a role in enhancing the inflammatory response. Hence, its downregulation is associated with cancer.

Chen et al. [128] found that A549 cells-derived exosomes were taken up by microglia HMC3 cells. Microglia are immune innate cells in the central nervous system and are reported to be enriched in brain tumors. These exosomes increase the phagocytic activity and secretion of pro-inflammatory cytokines IL-6, IL-8, and CXCL1 in microglia but do not affect their migration. The study also revealed that miR-1246 was highly expressed in exosomes derived from NSCLC cells and the plasma of NSCLC patients. Further experiments showed that microglia transfected with miR-1246 mimics exhibited increased secretion of IL-6, IL-8, and CXCL1, but no effect on proliferation was observed. These findings suggest that tumor exosomal miR-1246 may favor microglia’s production of inflammatory cytokines in vitro. In vivo experiments will further prove the observed effect on the central nervous system.

The literature review revealed that two tumor-derived exosomal miRNAs, miR-181b and miR-770, impact the polarization of macrophages toward the M2 phenotype, which is associated with promoting tumor growth. Exosomal miR-181b leads to the M2 phenotype and is upregulated in exosomes from NSCLC cells and serum from NSCLC patients. Conversely, miR-770 inhibits the M2 phenotype but is downregulated in exosomes from NSCLC cells and tumor tissue. Therefore, both exosomal miRNAs are linked to cancer promotion by influencing the M2 polarization. However, only miR-770 was proven to affect the growth of NSCLC tumors in vivo by obstructing M2 macrophage polarization. Meanwhile, exosomal miR-101 also affects macrophage function by suppressing IL1A and IL6 expression, which reduces growth and macrophage tumor infiltration in vivo. This miRNA is downregulated in tumor-derived exosomes by hypoxia, associated with tumor-promoting effects. Another exosomal miRNA, miR-574-5p, regulates pro-inflammatory PGE2 expression in tumor cells, but its impact on the immune response and tumor-related traits is untested. Finally, miR-1246 induces the increased expression of cytokines in microglia cells; however, its impact on the immune response and tumor-related mechanisms is untested. It is noted that the literature on exosomal miRNAs and the regulation of the immune response in lung cancer is still limited.

### 6.2. Regulation of Proliferation, Migration, Invasion, and Apoptosis

Cancer is characterized by continuous cell growth and division, which involves a complex interplay of genes and proteins, such as specific kinases and kinase receptors [129,130]. These components may become deregulated, allowing tumor cells to evade growth inhibition and enable sustained cell proliferation. Then, cancer cells must avoid programmed cell death or apoptosis to continue proliferating. Cell apoptosis involves extrinsic and intrinsic pathways that lead to the activation of caspases and, ultimately, cell destruction [130]. While evading the immune response, cancer cells can break through the stroma, migrate, and invade surrounding tissue. Several endogenous miRNAs have been implicated in promoting proliferation, migration, and invasion and inhibiting apoptosis in lung cancer [131,132]. In addition, evidence indicates that specific exosomal miRNAs derived from tumors promote lung cancer proliferation and migration while inhibiting apoptosis, as reviewed in the following literature.

A study conducted by Wu et al. [133] revealed that exosomes from H1299 lung cancer cells containing high levels of miR-96 promote the proliferation, migration, and drug resistance of A549 cells. In contrast, A549 cells transfected with a miR-96 inhibitor showed lower proliferation and migration and higher apoptosis levels, further supporting the regulatory role of miR-96. LIM-domain-only protein 7 (LMO7) is a target of miR-96, and its overexpression in A549 cells reversed the effects induced by exosomes from H1299 cells. The increased levels of miR-96 in both the tumor tissue and serum of lung cancer patients, along with the decreased level of LMO7 in tumor tissue, support the notion that miR-96 may play a tumor-promoting role in lung cancer.

According to Sun et al. [134], exosomal miR-106b promotes the migration and invasion of the NSCLC cell line SPC-A-1 while increasing MMP-2 and MMP-9 expression. This is achieved by exosomal miR-106b targeting the tumor suppressor PTEN, which promotes migration and invasion in SPC-A-1 cells. PTEN overexpression can reverse the effect of exosomal miR-106b on migration and invasion, further supporting its role in the regulatory mechanism. In patients with late-stage or lymph node metastases of lung cancer, the level of exosomal miR-106b in their serum was significantly higher, indicating a possible link between miR-106b and metastasis. However, additional in vivo studies are required to confirm the role of this miRNA in promoting metastasis.

On the other hand, altering the integrity of the vascular barrier facilitates the migration of cancer cells, leading to cancer spread. In this regard, Mao et al. [135] found that exosomes from miR-375-3p-transfected SCLC cells disrupt tight junctions in vascular endothelial cells by inhibiting the claudin-1 protein, breaking vascular barriers, increasing permeability, and promoting the transendothelial migration of SCLC cells in vitro. Experiments conducted in mice showed that injecting SCLS cells and exosomes carrying miR-375-3p resulted in increased metastatic nodules in the lung, indicating the potential role in metastasis. Accordingly, miR-375-3p was upregulated in the plasma of SCLC patients with metastasis from a small cohort, supporting its association with lung cancer metastasis.

In addition to miRNAs produced by tumor cells, miRNAs produced by other cell lineages have also been linked to mechanisms affecting lung cancer. For example, Lei et al. [136] discovered that exosomes released by THP-1 cells, which had differentiated into M2 cells, suppressed apoptosis and promoted the proliferation, migration, and invasion of A549 and SPC-A1 lung cancer cells. Exosomal miR-501-3p was upregulated in those M2 exosomes. They found that miR-501-3p mimics restricted apoptosis and facilitated the proliferation, migration, and invasion of A549 and SPC-A1 cells, whereas these effects were reversed by M2 exosomes downregulating miR-501-3p. The authors showed that WD repeat domain 82 (WDR82) was a target of miR-501-3p, and the overexpression of WDR82 impaired the growth of lung cancer cells induced by M2 exosomes. Their study suggests that M2-derived exosomal miR-501-3p facilitates the proliferation, migration, and invasion of lung cancer cells via targeting WDR82.

The literature review showed that two miRNAs, miR-96 and miR-106b, derived from tumors, and exosomal miR-375-3p, derived from M2 macrophages, can increase the proliferation, migration, and invasion of lung tumor cells. While these traits are associated with tumor progression, no in vivo studies were conducted to determine their impact on tumor progression or metastasis. On the other hand, SCLC-derived exosomal miR-375-3p, which breaks vascular barriers, increased metastatic nodules in the lung, indicating its potential role in metastasis. Nonetheless, more research is needed on this topic.

## 7. Exosomal miRNAs in Bone and Brain Metastasis

Around 30–40% of patients with late-stage NSCLC develop bone metastasis [137]. This type of metastasis can be classified as osteolytic, osteogenic, or mixed based on the radiographic and pathological features of the lesions [138]. Osteolytic metastasis results in bone destruction due to excessive osteoclast activity, while osteogenic metastasis is characterized by new bone deposition by osteoblasts [138,139]. Osteolytic bone metastasis is the most common type and accounts for 70% of all cancers, with most cases of NSCLC falling into this category [140]. In lung cancer patients with bone metastasis, the spine is the most frequently affected area, occurring in 40–50% of cases, followed by the ribs (20–27%) and pelvis (17–22%) [141,142,143]. Equally relevant, brain metastasis occurs in approximately 40% of NSCLC patients at diagnosis, while 25–40% will develop it during the disease [144,145]. In SCLC, 15% of patients have brain metastasis at diagnosis [146]. The most frequently affected areas are the cerebral hemispheres (80%), the cerebellum (15%), and the brainstem (5%) [147].

### 7.1. Bone Metastasis

Bone metastasis in lung cancer involves multiple steps, from survival in circulation to engagement and colonization of the bone compartment. During this process, bone tissue releases soluble factors, which may act as chemoattractants for receptors expressed in tumor cells, thereby aiding the process of metastatic homing [148]. For example, bone marrow stroma secretes C-X-C motif chemokine 12 (CXCL12), which attracts NSCLC tumor cells expressing its receptor C-X-C chemokine receptor 4 (CXCR4), directing tumor cells from blood circulation to the bone [149]. Afterward, the activation of multiple critical mechanisms and pathways is necessary for engagement within the bone microenvironment. Among them, inhibiting the collagen receptor discoidin domain receptor-1 (DDR1) in lung cancer cells hampers tumor cell survival, leading to impaired early tumor–bone engagement during skeletal homing. DDR1 is expressed in cancer cells and interacts with collagen in the stroma and bone marrow matrix [150]. The pathway involving the platelet-derived growth factor receptor (PDGFR) and vascular endothelial growth factor (VEGFR) plays a crucial role in the interaction between tumors and bone marrow stroma. PDGFR-β is expressed in the bone marrow stroma, and blocking it impairs the colonization of bone tissue in lung cancer [151,152]. Another pathway, the receptor activator of the nuclear factor-kappa β ligand (RANKL)/RANK, may be essential in mediating osteolytic bone destruction in bone metastasis of lung cancer [153,154]. Osteoclasts express RANKL, and RANK binding to RANKL stimulates osteoclast differentiation, survival, and activity, which may favor bone absorption balance [155,156]. Also, tumor cells produce several soluble factors, such as parathyroid hormone-related peptide (PTHrP), IL-5, and IL-11, which induce osteolysis by increasing RANKL expression [157,158].

In addition to the known mechanism of bone metastasis described above, evidence indicates that exosomal miRNAs have a role in lung cancer metastasis to the bone by facilitating osteoclastogenesis or affecting angiogenesis on site.

For example, Zhang et al. [159] found that miR-328-enriched exosomes from A549 cells enhanced RAW 264.7 osteoclastogenesis and osseous resorption in vitro and in vivo. In this study, murine osteoclast precursors RAW 264.7 transfected with miR-328 mimics showed decreased expression of osteoblast marker osteoprotegerin and increased staining of osteoclasts marker tartrate-resistant acid phosphatase (TRAP). These effects were reversed with miR-328 inhibitors, supporting the role of miR-328 in osteoclastogenesis. It was shown that miR-328 targets Neuropilin 2 (Nrp-2), and transfection of RAW 264.7 with Nrp-2 inhibitors enhanced TRAP staining. Notably, a deficiency of Nrp-2 has been previously associated with enhanced osteoclast differentiation and a reduced number of osteoblasts [160]. Further, in vivo experiments showed that mice treated with exosomes from A549 cells transfected with the miR-328 inhibitor had reduced trabecular bone thickness, increased trabecular separation, and lower osteoclast count. These findings indicated that exosomal miR-328 from A549 cells enhances osteoclastogenesis via downregulating Nrp-2.

In another study by Wang et al. [161], RAW264.7 cells were exposed to exosomes from bone metastatic NSCLC SPC-A-1BM or parenteral SPC-A-1 cells before being cultured under osteoclastogenesis conditions. RAW264.7 cells showed more significant differentiation into osteoclasts when exposed to exosomes derived from metastatic SPC-A-1BM cells compared to the parenteral cells. The authors found that the exosomes derived from SPC-A-1BM cells had higher levels of miR-17-5p than those derived from SPC-A-1 cells. They showed that miR-17-5p targeted PTEN, and the overexpression of PTEN inhibited the osteoclastogenesis of RAW264 cells caused by miR-17-5p transfection. Additionally, the osteoclastogenesis effects of miR-17-5p were effectively attenuated through inhibition of the PI3K/Akt pathway, suggesting that miR-17-5p promotes osteoclastogenesis through the PI3K/Akt pathway by targeting PTEN in NSCLC, which may be associated with osteolytic bone metastasis.

Xu et al. [162] reported that exosomal miR-21 from lung adenocarcinoma A549 cells also facilitates the osteoclastogenesis of murine bone marrow monocytes (BMMs) via targeting programmed cell death 4 (Pdcd4) in BMMs. Accordingly, researchers discovered that high levels of miR-21 in tumor tissue from adenocarcinoma patients predicted a poor prognosis, suggesting that miR-21 may promote metastasis.

On the other hand, Valencia et al. [163] found evidence that exosomal miR-192 may reduce the bone colonization of lung adenocarcinoma cells by inhibiting angiogenesis at the site. In this study, miR-192 was downregulated in highly metastatic subpopulations (HMS) derived from A549 cells with a tendency to form osseous metastases. Exosomes from miR-192-transfected HMS cells impaired vessel connectivity in HUVEC cells, suggesting anti-angiogenic effects. However, they did not affect osteoclastogenesis assays in vitro. In vivo, assays showed that mice treated with exosomes from HMS cells had increased osteolytic lesions than those treated with exosomes from A549 parenteral cells. The effect was reversed with exosomes derived from miR-192-transfected HMS cells. This indicates that exosomal miR-192 may reduce bone colonization by decreasing on-site angiogenesis, explaining its low expression in highly metastatic HMS cells.

Noticeably, the revised literature revealed no links between exosomal miRNAs and most cytokines, growth factors, molecular receptors, or pathways commonly associated with previously known mechanisms of bone metastasis. We acknowledge the importance of distinguishing between these general mechanisms, as described in the background text, and those explicitly related to exosomal miRNAs, which may still contribute to osteoclastogenesis. Most importantly, this indicates the need for additional research rather than a lack of connection between miRNA-regulated and known bone metastasis mechanisms. Table 3 summarizes the exosomal miRNAs associated with osteoclastogenesis in lung cancer.

### 7.2. Brain Metastasis

The progression of lung cancer that spreads to the brain involves several critical stages, including survival in the bloodstream, infiltration of the blood–brain barrier (BBB), and invasion of the central nervous system (CNS) [164]. The BBB comprises endothelial cells with tightly bonded junctions that line cerebral microvessels. It is a selective barrier that allows nutrients to flow through and restricts ionic and fluid movements while blocking toxins in the blood from entering the brain [165]. BBB remodeling has been observed in brain metastasis, which may facilitate the entrance of tumor cells. For example, in experimental brain metastases and surgical samples of human lung cancer, blood vessels associated with brain metastasis are dilated and have many dividing endothelial cells. Also, tumor cells may release the vascular endothelial growth factor (VEGF), which causes permeability in nearby vessels. During transendothelial migration in SCLC, Rho GTPases become activated, which leads to an increase in actomyosin contractility and facilitates the breakdown of intercellular junctions [166,167]. Additionally, tumor cells secrete growth factors, metalloproteinases, and proteases to migrate through the BBB, such as prostaglandin-endoperoxide synthase 2 (COX2), the heparin-binding EGF-like growth factor (HB-EGF), matrix metalloproteinases (MMPs), and cathepsin S [168]. Once tumor cells have reached the brain parenchyma, they must survive and adapt to the new microenvironment, which will depend on the complex interactions with resident cells such as astrocytes, microglia, and neurons. For example, lung tumor cells produce serpins that act as anti-plasminogen activators (PAs), which shield them against the pro-apoptotic impact of PAs secreted by astrocytes [169]. Tumor cells induce astrocytes and microglia to express endothelin-1 (ET-1), which activates survival and chemo-resistance signals in the tumor cells [170].

Endogenous miRNAs have a significant impact on the development of brain metastasis in lung cancer, especially in NSCLC. They are involved in various processes, including EMT, migration, colonization, the regulation of stemness, and the creation of a conducive environment for metastasis in the brain [171,172,173,174,175,176]. However, there is a significant gap in research on the role of exosomal miRNAs in the progression of brain metastasis in lung cancer, as evidenced by the available information.

Wei et al. [177] found that exosomes from the plasma of lung cancer patients with brain metastasis (n = 3) contain higher levels of miR-550a-3-5p than those from patients without it (n = 3). They observed that human brain microvascular endothelial cells (HBMECs) treated with exosomes from metastatic patients and a high metastatic lung cancer cell line (95D) showed significantly lower viability and migration, higher cell apoptosis, and reduced number of cells during the G0/G1 phase compared with non-metastatic controls. Similar effects were obtained after transfection with miR-550a-3-5p mimics on HBMECs, whereas the opposite effects were observed with miR-550a-3-5p inhibitors. The authors found that Yes1-associated transcriptional regulator (YAP1) is a target for miR-550a-3-5p. YAP1 is a protein that regulates the transcription of genes involved in cell proliferation, apoptosis, homeostasis, and repair through the Hippo signaling pathway. It is linked to several cancer developments and could be a target for cancer treatment [178]. These findings suggest that exosomal miR-550a-3-5p may regulate cell viability, apoptosis, the cell cycle, and the migration of HBMECs via YAP1; however, additional research is necessary to determine the effects on lung cancer-associated brain metastasis.

**Figure 3:**
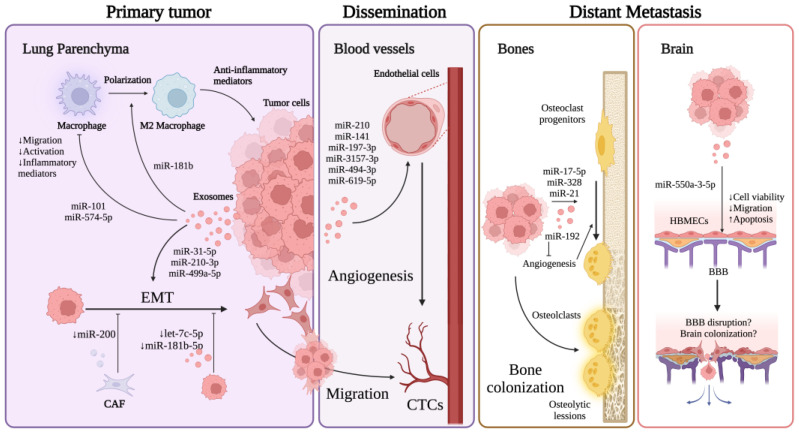
Exosome-derived microRNAs associated with lung cancer metastasis progression. Exosomal miRNAs impact the spread of lung cancer at the primary tumor site by regulating processes such as EMT, angiogenesis, and the immune response. While studies indicate that exosomal miRNAs can either promote or inhibit these biological processes, their levels in cancer tissue ultimately suggest a promoting effect on metastasis. Not only do these exosomal miRNAs impact the spread of cancer locally, but they also affect the development of secondary tumors in distant areas. Specifically, bone metastases can promote the creation of osteoclasts, and brain metastases can disrupt the blood–brain barrier, thus facilitating tumor cell colonization. BBB, blood–brain barrier; EMT, epithelial-to-mesenchymal transition; TME, tumor microenvironment. Created with BioRender.com, “https://www.biorender.com/ (accessed on 4 September 2023)”.

Importantly, although in breast cancer, Tominaga et al. [179] reported that brain metastatic breast cancer cells release microRNA-181c-containing extracellular vesicles (EVs) capable of destroying the blood–brain barrier. They showed that miR-181c promotes the destruction of the BBB through the abnormal localization of actin via the downregulation of PDPK1. PDPK1 degradation downregulated phosphorylated cofilin and the resultant activated cofilin-induced modulation of actin dynamics. Moreover, the systemic injection of EVs derived from brain metastatic cancer cells promoted brain metastasis of breast cancer cell lines and were preferentially incorporated into the brain in vivo. This revealed a novel mechanism mediated by EVs that triggers the destruction of the BBB, which facilitates brain metastasis. Figure 3 summarizes the exosome-derived microRNAs associated with lung cancer metastasis progression.

## 8. Conclusions and Perspectives

Understanding the mechanisms of metastasis is a crucial area of research in the biomedical field. However, this is not a simple task. Tumor metastasis is a complex process involving the activation of multiple mechanisms and pathways, as well as complex communication signals between resident stromal, immune, and cancer cells. This communication occurs through direct contact between cells or via secreted factors that can be transported by small extracellular vesicles like exosomes. Tumor cell exosomes play a significant role in metastasis progression by releasing biologically active components, such as miRNAs, to local and distant sites, stimulating tumorigenesis, and preparing the tumor pre-niche. Indeed, recent research has linked exosome-derived microRNAs to mechanisms of metastasis progression in lung cancer.

This review’s updated literature sheds light on the exosomal miRNAs’ identity that plays a role in lung cancer metastasis. It also examines the donor and recipient cells and their molecular mechanisms and targets. The studies indicate that current research on lung cancer metastasis is still in its early stages. However, they emphasize the importance of comprehending how exosomal microRNAs impact metastasis to develop new targeted treatments in the future.

These studies have identified specific exosomal miRNAs regulating critical mechanisms and their targets in lung cancer metastasis (Table 1, Table 2 and Table 3). Hypothetically, if we understand how exosomes use miRNAs to promote metastasis, we may be able to stop this process. However, it is important to note that the same miRNAs regulate different targets, and multiple miRNAs regulate the exact mechanisms through the same or different targets. This is expected due to the miRNAs’ multi-target and redundant regulatory capacities. Moreover, exosomes that carry miRNAs have surface receptors and molecular cargo that vary in composition, likely depending on the type and physiological state of donor cells (Section 2). Further, the mechanisms of how and which miRNAs are selectively loading into exosomes are still under study (Section 2).

We must emphasize that the nature and function of exosomes make this research field highly interesting and challenging. How these exosomes prepare a pre-metastatic niche in nearby and distant organs through clinically undetectable mechanisms during cancer is critically relevant. Stocking this process before the cancer cells reach and progress in a new metastatic site is a highly desirable objective.

Hence, identifying specific exosome-derived miRNAs, their molecular targets, and mechanisms involved in metastasis progression is an early but critical research stage toward clinical applicability. The next step is demonstrating that inhibiting these factors can hinder metastasis progression. This process starts with in vivo experiments in the laboratory and advances to the clinical level for new targeted therapies.

There are only a small number of in vivo studies focusing on lung cancer metastasis and exosomal miRNAs. Exosomal miR-31-5p and miR-499a-5p, associated with EMT, affect tumor progression in murine models (Table 1). Additionally, exosomal miR-210, miR-141, and miR-197-3p, associated with angiogenesis, induce increased microvessel density and tumor growth in murine models (Table 2). Some studies found that the expression of certain tumor-exosomal miRNAs associated with metastasis correlates with levels of circulating or tissue miRNA in patients with metastasis and poor prognoses. These miRNAs include miR-197-3p, miR-3157-3p, miR-619-5p, miR-770, miR-1246, miR-21, and miR-550a-3-5p. This suggests that these specific miRNAs are relevant in the disease progression of lung cancer patients and could potentially be used as biomarkers for clinical applications.

Based on the revised literature, we conclude that more research is needed to fully understand the role of exosome-derived miRNAs in the metastasis of lung cancer. It is important to note that overcoming current technical limitations will be critical for progress in this field. Specifically, we need to develop standardized methods for isolating defined subpopulations of EVs, such as exosomes, as discussed in previous works [33]. Additionally, it will be challenging to devise effective ways to study the transport, delivery, and uptake of exosomal miRNAs [33]. The lack of current clinical trials for exosomal miRNAs as a targeted therapy in lung cancer reflects that this is still an early-stage area of research, with much work still needed in vivo. By overcoming the technical challenges and performing more research on exosome-derived miRNAs in lung cancer, investigators can better understand their biological significance. Furthermore, they can explore the potential of using miRNAs within exosomes or EVs as therapy tools to specifically target organs affected by metastasis, improving the effectiveness of treatment. These advancements will enable researchers to pursue personalized cancer treatments.

## Figures and Tables

**Table 1:** Exosomal miRNAs associated with EMT in lung cancer.

Exosomal miRNA	Cellular Source	Recipient Cells	Molecular Targets	Possible Mechanism	Effects	Reference
EMT promoting						
miR-31-5p	A549 H1299	A549 H1299	SATB2	MEK/ERK signaling activation	↑ Migration, invasion, EMT, and tumor progression	[87]
miR-210-3p	A549	A549 NCI-H1703	FGFRL1	Unknown	↑ Migration, invasion, EMT, and MMP-9/MMP-1 expression.	[88]
miR-210-3p	HCC827-OR	HCC827 (parental cells)	Unknown	Unknown	↑ EMT and resistance to Osimertinib	[89]
miR-499a-5p	SPC-A-1BM	SPC-A-1 (parental cells)	S6K1, BP1	mTOR pathway activation	↑ EMT, proliferation, and migration. Larger tumor nodules	[90]
EMT inhibiting						
miR-200	CAF	A549 NCI-H460	ZEB1	Unknown	Inhibition of migration, invasion, and EMT	[95]
let-7c-5p and miR-181b-5p	A549	BEAS-2B	Unknown	MAPK signaling pathway?	Inhibition of migration, invasion, and EMT	[96]

BP1, eukaryotic initiation factor 4E-binding protein 1; CAF, cancer-associated fibroblasts; EMT, epithelial-to-mesenchymal transition; ERK, extracellular signal-regulated kinase; FGFRL1, fibroblast growth factor receptor-like 1; MAPK/MEK, mitogen-activated protein kinase; MMP-1, matrix metallopeptidase 1; MMP-9, matrix metallopeptidase 9; mTOR, mammalian target of rapamycin; PFD, pirfenidone. S6K1, S6 kinase beta-1; SATB2, special AT-rich sequence-binding protein 2; ZEB1, zinc finger E-box binding homeobox 1; ↑, increased.

**Table 2:** Exosomal miRNAs associated with angiogenesis in lung cancer.

Exosomal miRNA	Cellular Source	Recipient Cells	Molecular Targets	Possible Mechanism	Effects	Reference
miR-210	A549 H460	NIH/3T3	TET2	STAT3/JAK2 signaling activation	Promotion of CAF phenotype. ↑ Secretion of pro-angiogenic factors VEGF-A, FGF2, and MMP9 by CAF. ↑ Proliferation, migration, and tube formation of endothelial cells. ↑ In vivo microvessel density.	[105]
miR-141	H466 H1048	HUVEC	KLF12	Unknown	↑ In vivo microvessel density.	[108]
miR-141	A549	HUVEC	GAX	Unknown	↑ Formation of tubes of endothelial cells.	[112]
miR-197-3p	A549 H1299	HUVEC	TIMP2, TIMP3	Unknown	↑ In vivo tumor growth	[114]
miR-3157-3p	A549 H1299	HUVEC	TIMP2, KLF2	Unknown	↑ Expression of VEFG, MMP2, and MMP9. ↑ Vascular permeability.	[115]
miR-494-3p	A549	HUVEC	PTEN?	cJun/PTEN pathways	↑ Formation of tube formation	[116]
miR-619-5p	A549	HUVEC	RCAN1.4	Unknown	↑ In vitro invasiveness of A549 cells ↑ In vitro tumor expression of CD31	[117]

CAF, cancer-associated fibroblasts; GAX, growth arrest-specific homeobox gene; KLF12, Krueppel-like factor 12; MMP-2, matrix metallopeptidase 2; MMP-9, matrix metallopeptidase 9; PTEN, phosphatase and tensin homolog; RCAN1.4, regulator of calcineurin 1 isoform 4; TET2, ten-eleven translocation 2; TIMP2, tissue inhibitor of metalloproteinase 2; TIMP3, tissue inhibitor of metalloproteinase 3; ↑, increased.

**Table 3:** Exosomal miRNAs associated with osteoclastogenesis in lung cancer.

Exosomal miRNA	Cellular Source	Recipient Cells	Molecular Targets	Possible Mechanism	Effects	Reference
miR-17-5p	SPC-A-1BM	RAWR264.7 cells	PTEN	Inhibition of PI3K/Akt pathway	Unknown	[161]
miR-328	A549	RAWR264.7 cells	Nrp-2	Unknown	Unknown	[159]
miR-192	A549	HMS	Unknown	Unknown	↓ In situ angiogenesis	[163]
miR-21	A549	BMM	Pdcd4	c-Fos inhibition	Unknown	[162]

BMMs, bone marrow monocytes; HMS, highly metastatic subpopulations; Nrp-2, neuropilin 2; Pdcd4, programmed cell death 4; PI3K/Akt, phosphatidilinositol 3-kinase/protein kinase-B; PTEN, phosphatase, and tensin homolog; ↓, decreased.
